# Occupational leg edema—use of compression stockings

**DOI:** 10.1097/j.pbj.0000000000000093

**Published:** 2020-11-24

**Authors:** Pedro M. Guedes, Nuno A. Saldanha, Pedro M. Matos, Francisco S. Carvalho, Graça Veiga, Pedro Norton

**Affiliations:** Centro Hospitalar Universitário São João, Serviço de Saúde Ocupacional, Porto, Portugal

**Keywords:** compression stockings, occupational health, occupational leg edema, preventive medicine

## Abstract

To analyze the use of compression stockings to avoid the formation of occupational edema of the lower limbs in jobs with prolonged orthostatism. We carried out a review of the articles published in PubMed, from the 1st of January 2008 to 31st of December 2018 using the term “Occupational Leg Swelling”. Only articles that met the following criteria were selected: prospective, observational and experimental retrospectives articles written in Portuguese or English. The research resulted in 23 articles. After reading the titles and abstracts and applying the inclusion and exclusion criteria, 5 were selected. Prolonged orthostatism is considered a risk factor for the development of chronic venous disease. The use of compression stockings reduces the occupational edema of the lower limbs. Professionals exposed to prolonged orthostatism during their work activity have a higher risk of developing lower limb edema; Despite few studies demonstrated the effectiveness of wearing compression stockings to prevent occupational edema of the lower limbs, they have showed benefit in reducing edema as well as associated symptoms. The use of compression stockings should therefore be recommended to all professionals at increased risk for occupational edema of the lower limbs.

## Introduction

Occupational edema of the lower limbs is a very common complaint among workers exposed to prolonged orthostatism. Most cases are asymptomatic, but sometimes they are associated with “heaviness” and diffuse pain in the lower limbs.^1^ Long periods of orthostatism cause a decrease in venous return, increasing the risk of chronic venous disease (CVD)^2,3^ and the associated symptoms.^4^ There are few studies that investigate the effectiveness of using compression stockings to prevent CVD, in professionals exposed to prolonged orthostatism.

## Methods

A systematic review was carried out on PubMed (Medline), with the MeSH Term “occupational leg swelling” between the 1st of January of 2008 and the 31st of December of 2018. Articles were selected by title and abstract in Portuguese and English. Prospective articles, observational and experimental retrospectives were included and non-original meta-analyzes and studies (reviews, letters from authors and editorials) were excluded. The relevant data for the research (authors, year of publication, description of the sample, main conclusions) are shown in Table 1.

**Table 1:** Articles that resulted from the research and main conclusions

Authors	Year of publication	Sample	Objectives	Conclusions
Belczak et al^5^	2008	70 Health professionals	Compare which work period is associated with greater occupational edema	In professionals who work 12 hours continuously, the increase in leg volume is significant, regardless of the period of the day. The rate of edema formation is higher in the morning shift
Blazek et al^6^	2013	98 Hairdressers	To evaluate the impact of wearing compression stockings on occupational edema of the lower limbs	Both groups showed decreased leg volume with the use of compression stockings. With the cessation of compressor treatment, symptoms reappear
Mosti et al^7^	2013	30 Health professionals	Compare progressive compression stockings with graduated compression stockings in reducing occupational edema	Both socks significantly reduce occupational edema, but the progressive ones show a greater reduction and not only of specific area
Diken et al^8^	2016	232 Nurses	To determine the prevalence of CVD in nurses subjected to periods of prolonged orthostatism	Changes in working conditions can contribute to the improvement of CVD symptoms; The time spent in hospital hours is associated with a higher frequency of signs of CVD symptoms
Wou et al^9^	2016	10 Health professionals	Compare the use of compression stockings with neuromuscular stimulation devices	Compression stockings were the only method that effectively reduced lower limb edema

## Results

The initial sample consisted of 23 articles, 7 of which were selected after analyzing the titles and abstracts. After applying the defined criteria, the final sample consisted of 5 articles.

## Discussion

It was consensual, for the different authors, that the professionals exposed to prolonged orthostatism or sedentary lifestyle at work had more risk for developing edema of the lower limbs.^5–9^ Diken et al^8^ studied the prevalence of CVD in nurses exposed to occupational risk factors, especially periods of prolonged standing and found a positive correlation between the mean hours of work and the formation of edema and associated symptoms. The same authors reported that, professionals who worked in services with a more prolonged length of stay had less edema occupational. This situation could be explained by a more efficient management of daily tasks, and a more adequate control of the occupational risks. In opposition, services with a shorter hospital stay length have a increased turnover of patients associated with greater physical demand and less control over the rhythm of the tasks performed which would explain the results. Belczak et al^5^ studied the variation in occupational edema in healthcare professionals to understand if there was a relationship between edema and working periods. Three groups of healthcare professionals (group I—morning shift; group II—afternoon shift; group III—12-hour continuous period). In all groups there were significant variations in the perimeter of the lower limbs. Installation speed edema was more evident in the morning shift. The pathophysiology that could justify this variation in perimeter is due to the fact that there is a stabilization of pressures at the level of the microcirculation counterbalancing the intravascular pressure and extravascular during the early hours of the morning. Due to gravitational forces, the legs tend to have a higher rate of edema formation at this period of the day with an increase in interstitial pressure that interferes with tissue overflow seem throughout the day. The authors defended the need to adopt preventive measures, namely the use of compression stockings to reduce this occupational edema in professionals with known risk. Blazek et al^6^ carried out a study in a population of hairdressers. It was evaluated whether the use of low compression stockings grade (grade I) decreases occupational edema and associated symptoms. According to the disease classification system CVD, 83% of participants were in a C0 stage (No signs of sensitive venous diseases or palpable) or C1 (Telangiectasias or reticular veins). There was no association between the reduction of occupational edema and the prevalence of symptoms; in some cases the edema remained, but there was a reduction in symptoms. The participants, when they stopped wearing compression stockings, reported the onset of symptoms. The authors defended that the use of compression stockings could not decrease the edema, but cause symptomatic relief, namely pain, and the feeling of heavy legs. Mosti et al^7^ studied the different types of compression stockings, progressive and graduated. Both types edema significantly decreased, but progressive ones seem to have better results. The authors defended the need to assess whether progressive stockings would be associated with the formation of edema in the “gaiter area” as a preferred place for the occurrence of skin changes and ulcers. Wou et al^9^ evaluated the effectiveness of neuromuscular stimulation as an alternative method to the use of compression stockings. Despite the small sample size (n = 10), the study showed that compression stockings were the only method that effectively decreased lower limb edema. In addition, compression stockings were the preferred method for participants when asked about the effectiveness of the treatment. Prolonged orthostatism as well as sedentary work are extremely important risk factors for edema occupational.^10^ Despite the few studies carried out in this area, the use of low compression stockings (grade I) seems to be the most effective method in preventing occupational edema as well as associated symptoms. This review showed the need for further studies to assess risk factors for occupational edema, namely organizational risk. Physicians should encourage the use of compression stockings in workers exposed to periods of prolonged orthostatism in order to prevent the development of CVD.

## Acknowledgments

None.

## Financial support

None.

## Conflicts of interest

The authors declare no competing interests.
